# European Working Group on Sarcopenia in Older People Algorithm: Step‐by‐Step Relation With Length of Hospitalization

**DOI:** 10.1111/jgs.70312

**Published:** 2026-02-05

**Authors:** Elena Zoico, Silvia Urbani, Anna Giani, Francesco Fantin, Alessandro Gavras, John A. Batsis, Rocco Micciolo, Mauro Zamboni

**Affiliations:** ^1^ Division of Geriatric Medicine, Department of Medicine University of Verona Verona Italy; ^2^ Centre for Medical Sciences‐CISMed, Department of Psychology and Cognitive Science‐DIPSCO, Section of Geriatric Medicine University of Trento Rovereto Italy; ^3^ Division of Geriatric Medicine, School of Medicine, and Department of Nutrition, Gillings School of Global Public Health University of North Carolina at Chapel Hill School of Medicine Chapel Hill North Carolina USA; ^4^ Centre for Medical Sciences‐CISMed, Department of Psychology and Cognitive Science‐DIPSCO University of Trento Rovereto Italy

**Keywords:** aging, body composition, dynapenia, SARC‐F, sarcopenia

## Abstract

**Background:**

An international consensus is still lacking on the best operational definition of Sarcopenia in hospitalized older adults. The main objective of this study was to use the EWGSOP2 guidelines in hospitalized old subjects to test its predictivity for adverse clinical outcomes and to evaluate its step‐by‐step capability to predict unfavorable clinical events.

**Participants and Setting:**

Three hundred and seventeen men and two hundred and eighty seven women, aged 65 to 99 years, consecutively admitted to the Department of Geriatrics at the University Hospital of Verona.

**Methods:**

All patients underwent a complete geriatric assessment, clinical evaluation, and for the diagnosis of Sarcopenia, the EWGSOP2 guidelines were applied. As clinical outcomes, length of hospital stay, fall risk, and subjects' quality of life were considered.

**Results:**

Among 604 hospitalized older patients, 56.0% presented with a SARC‐F score suggestive of a risk for Sarcopenia. Patients at risk for Sarcopenia, and with available handgrip strength data, in 85.5% of cases also presented probable Sarcopenia. Among patients with probable Sarcopenia, and with available body composition data, 83.1% were confirmed with Sarcopenia, with a general prevalence of Sarcopenia of 22%. The shortest average length of hospitalization was in non‐sarcopenic patients, with a median of 11 days, whereas dynapenic and sarcopenic subjects have respectively a median of 12 and 13 days of hospitalization, with significant differences also after adjustment for age, nutritional status and comorbidity. After dividing the patients into negative or positive for each diagnostic step of the EWGSOP2 algorithm, we found, for each step of the algorithm, a progressively greater association with adverse clinical outcomes.

**Conclusions:**

EWGSOP2 algorithm is a valid tool even in hospitalized older patients, and each step enhances the predictivity of the algorithm; however, SARC‐F and muscle strength can still be valuable tools for negative clinical outcomes when body composition data are not available.

## Summary

- Key points
  ○ Our findings indicate that the EWGSOP2 algorithm is a reliable instrument even for hospitalized older patients.
  ○ Our results show that each step of the EWGSOP2 algorithm improves predictive validity.
  ○ SARC‐F and muscle strength may be useful indicators for adverse clinical outcomes when body composition data are unavailable in hospitalized older patients.
- Why does this paper matter?
  ○ These data could be helpful in finding a good operational definition for Sarcopenia, also in populations of hospitalized frail and disabled older subjects.
  ○ Finding a good operational definition for Sarcopenia could be made easier by these data, particularly in populations of older hospitalized frail patients.

## Graphical abstract

This study globally shows that the EWGSOP2 algorithm is a valid tool even in hospitalized older patients. In fact, every step enhances the predictive validity of the algorithm, suggesting, however, that SARC‐F and muscle strength can still be valuable tools for negative clinical outcomes when body composition data are not available.

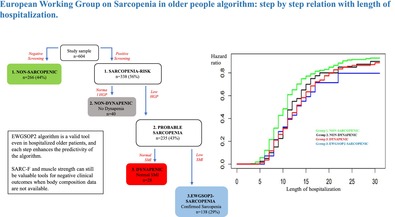

## 1 Introduction

Since the first identification of Sarcopenia [1], several definitions of this condition, characterized by an age‐related loss of muscle mass, strength, and function, have been proposed by different authors, working groups, and institutions [2, 3]. However, despite a considerable increase in Sarcopenia research in the past few decades [4], consensus on the best operational definition of Sarcopenia is still lacking [5]. Depending on the definition used and on the population considered, the prevalence of Sarcopenia ranges from very low to considerably high rates of occurrence [3, 6, 7]. Stuck and colleagues [8], tested different definitions of Sarcopenia in a population of 1495 old European subjects of the DO‐HEALTH study, finding a Sarcopenia prevalence ranging from 0.7% using the EWGSOP2 definition, up to 16.8% using the Delmonico one.

In a recent Delphi survey, conducted by the Global Leadership Initiative on Sarcopenia (GLIS), the key findings in the conceptual definition of Sarcopenia were muscle mass, muscle strength, and muscle‐specific strength [9]. However, how to translate this conceptual definition into an operational one still remains a challenge for the next future international initiatives.

Moreover, the current definitions of Sarcopenia were more often used in outpatient settings and only rarely in studies enrolling hospitalized older patients who are subject to a severe loss in physical function and in basic activities of daily living [6, 10]. In fact, only a few studies provide data about the prevalence and outcomes of Sarcopenia among hospitalized older individuals [6, 10, 11]. In 305 old hospitalized patients, aged 65 years and older, the prevalence of probable and confirmed Sarcopenia, according to the EWGSOP2 definition, was respectively 24.6% and 22.6%, [11], in line with previous reports [12, 13]. The question whether different methods and/or cut‐off values should be used for the operational diagnosis of Sarcopenia in older frail populations is still unanswered even though, according to the recent GLIS consensus, the conceptual definition of Sarcopenia should not vary by setting of care, by age, or health conditions [9].

The present study attempted to explore these uncertain points in Sarcopenia research. In particular, the main objective of this study was to use the EWGSOP2 guidelines in a population of hospitalized older subjects of both sexes to test its predictivity for adverse clinical outcomes. Second, we evaluated the step‐by‐step predictivity of the algorithm to show and compare the capability of each step to predict unfavorable clinical outcomes.

## 2 Methods

### 2.1 Study Population

Subjects included in this study were recruited from old patients consecutively admitted to the Department of Geriatrics at the University Hospital of Verona, Verona, Italy. The only inclusion criteria considered were age 65 years and older, and SARC‐F questionnaire completed by the patient or the caregiver. In total, 317 men and 287 women aged 65 to 99 years were recruited. The study was approved by the Ethical Committee of the Verona University Hospital and it was conducted in accordance with the latest revision of the Helsinki Declaration. A written consent to participate in the study was signed by all the subjects or by the proxy caregiver in patients unable to provide it.

### 2.2 Comprehensive Geriatric Assessment

All patients underwent a complete clinical evaluation, comprehensive of detailed clinical history, pathological conditions, previous admissions, and drug regimen.

Pre‐admission functional abilities were based on the last week before admission, either by self‐report by the patient or their caregiver. Activities of Daily Living (ADL) were assessed according to Katz [14] and Instrumental ADL according to Lawton [15]. Barthel Index [16] was used to assess functional independence in the domains of daily self‐care activities and mobility. Comorbidity and Frailty status were assessed respectively by the Charlson Comorbidity Index (CCI) [17] and the Rockwood Clinical Frailty Scale [18]. Nutritional status was evaluated with the Mini‐Nutritional Assessment (MNA) form [19]. For outcomes analysis, fall risk was assessed by using the Hendricks Fall Risk Score [20] and subjects' quality of life with a visual analogical scale (QoL‐VAS = quality of life Visual Analog Scale) [21]; the length of hospitalization in the Geriatric Department was also recorded.

Body weight and height were measured at admission in all subjects, and Body Mass Index (BMI) was calculated as weight (kg)/height2 (m2) [22]. Where body height was not possible to measure, we used the estimated height based on the knee height [23].

Biochemical evaluation was obtained in all subjects with a full panel of laboratory tests as per routine clinical practice.

### 2.3 Sarcopenia Diagnosis

**FIGURE 1:**
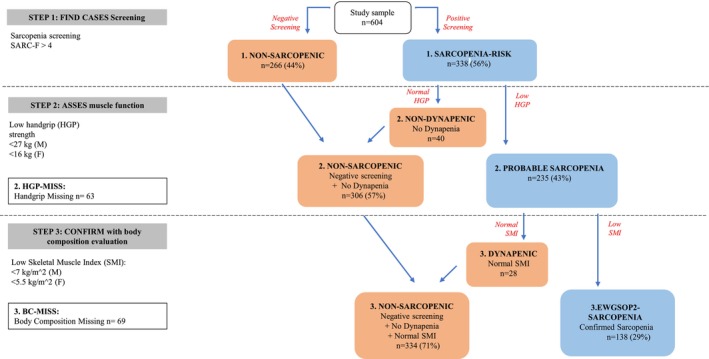
Step‐by‐step application of the EWGSOP2 algorithm and identification of different groups of hospitalized patients (*n* = 604).

For the diagnosis of Sarcopenia, the steps indicated by the EWGSOP2 algorithm were applied sequentially [24]. Figure 1 represents all the steps used for the diagnosis of Sarcopenia.

In the first step (STEP 1), all enrolled old subjects (*n* = 604) were administered the SARC‐F questionnaire to screen for the risk of Sarcopenia. A score of ≥ 4 on the SARC‐F questionnaire indicates a risk of Sarcopenia [25]. Only patients with SARC‐F ≥ 4 underwent assessment of muscle strength (STEP 2) by handgrip dynamometer (Jamar Handheld Dynamometer) [24]. The best of three trials with the dominant hand was considered for the analysis, using the EWGSOP2 guidelines cut‐off values [24]. Patients who had no reliable or available measurement of handgrip strength were excluded (*n* = 63: 50% severe dementia, 40% critical illness, 1.6% hemiplegia and 8.4% missing data).

In the last step (STEP 3), only in patients with probable Sarcopenia (*n* = 235) (i.e., SARC‐F ≥ 4 and handgrip suggestive of Dynapenia), muscle mass was measured by a portable multi‐frequency impedentiometer, (BIA 101 BIVA PRO, AKERN, Firenze, Italy). This instrument was used at a single frequency of 50 kHz to analyze several parameters. Appendicular Skeletal muscle Mass (ASM) was calculated using the equation by Sergi and colleagues [26] and the EWGSOP2 cut‐off points used [24]. In 69 patients, body composition data were missing due to unreliable measures (51% cardiac fluid overload, 12% fluid therapy for critical illness, 10% ascites and 4.3% bilateral hip prosthetic replacement) or to absent data (22.7%). Sarcopenia was confirmed in 138 patients (i.e., the presence of low muscle strength combined with low muscle mass according to the EWGSOP2 criteria [24]).

### 2.4 Statistical Analysis

For outcomes analyses the study population was first stratified into 4 groups (Figure 1). *Group 1 NON‐SARC (Non Sarcopenic subjects)* includes patients with a negative screening test by SARC‐F, (not evaluated in the next steps); *Group 2 NON‐DYNAP (Non Dynapenic subjects)* comprises patients with a positive screening test by SARC‐F, but with normal handgrip strength (not evaluated in the next steps); *Group 3 DYNAP (Dynapenic subjects)* includes older subjects with a positive screening test, low handgrip measures but preserved muscle mass; *Group 3 EWGSOP2‐SARC (Confirmed Sarcopenia)* comprises patients with either SARC‐F, handgrip strength test and SMI values, compatible with Sarcopenia diagnosis by EWGSOP2 guidelines [24]. We excluded from the analyses *Group 2 HGP‐MISS (Handgrip missing)* and *Group 3 bc‐MISS (Body composition missing)* where BIA evaluations were not available (see Table S1).

Finally, to test outcomes predictivity of each step of the EWGSOP2 algorithm, we divided the patients into negative or positive for each diagnostic step of the EWGSOP2 flowchart and performed outcome analyses for each step, comparing *group 1. NON‐SARC* versus *group 1. SARC‐RISK*, *group 2. NON‐SARC* versus *group 2. PROB‐SARC* and *group 3 NON‐SARC* versus *group 3. EWGSOP2‐SARC* (Figure 1).

To evaluate the differences in means between the four groups under examination, the Analysis of Variance (ANOVA) was employed, while comparisons taking into account the effect of selected covariates were performed by means of Analysis of Covariance (ANCOVA).

Length of hospitalization was analyzed employing the product limit estimator [27], while the log‐rank test [28] was used to compare length of hospitalization among different groups. The Cox model and the likelihood ratio test were employed to estimate the hazard ratio of discharge before and after adjustment for selected covariates [29]. All the analyses were performed employing the R software (*version 4.2.0*) [30]. The results were presented as mean ± standard deviation (SD). A significance level of 5% was adopted.

## 3 Results

The analysis included 604 hospitalized older patients admitted to the Department of Geriatrics, with a mean age of 85.3 ± 6.4 years, and for the 52.5% represented by male subjects. The group of female patients was significantly older compared to that of male patients, with comparable mean BMI values between the groups; ADL, IADL, and the Rockwood Clinical Frailty Scale presented worse scores in older women compared to older men, despite no significant difference in the MNA and in the CCI (Table S2). Moreover, the main outcomes of the study were not significantly different between the two groups of older female and male patients (Table S2).

In this group of 604 old patients, we first applied sequentially the EWGSOP2 algorithm for the diagnosis of Sarcopenia [24], stratifying the enrolled population into different groups (Figure 1). In STEP 1, 338 subjects (56.0%), presented a SARC‐F score > 4, suggesting a risk for Sarcopenia (Figure 1). In STEP 2, among the 275 subjects identified at risk for Sarcopenia and with available handgrip dynamometer data, 235 old subjects (i.e., 85.5%) presented with Dynapenia and thus probable Sarcopenia (Figure 1). In STEP 3, using the EWGSOP2 algorithm, among the 166 patients with probable Sarcopenia, and with available body composition data, 138 (i.e., 83.1%) were confirmed as affected by Sarcopenia (Figure 1).

The main characteristics of the groups identified according to the EWGSOP2 guidelines are presented and compared in Table 1. Subjects with confirmed Sarcopenia and with Dynapenia were older, more disabled in ADL and IADL scale and presented a higher comorbidity at the CCI, compared to subjects with a negative screening for Sarcopenia or with a positive screening for Sarcopenia but without Dynapenia (Table 1). BMI and serum albumin mean levels were tendentially lower in patients with confirmed Sarcopenia, compared to the other groups (Table 1). MNA and the Rockwood Clinical Frailty Scale presented better scores in older subjects of the group with a negative screening for Sarcopenia compared to all the other groups (Table 1).

**TABLE 1:** General characteristics of the study populations of old patients stratified in different groups according to the application of the EWGSOP2 algorithm for Sarcopenia screening and diagnosis (*n* = 604).

	Group 1. NON‐SARCOPENIC Negative Screening (*n* = 266)	Group 2. NON‐DYNAPENIC Positive Screening, normal handgrip (*n* = 40)	Group 3. DYNAPENIC Dynapenia, normal SMI (*n* = 28)	Group 3. EWGSOP2‐SARCOPENIC Sarcopenia (*n* = 138)		
	Mean ± SD	Mean ± SD	Mean ± SD	Mean ± SD	Raw	Age‐adjusted
Age (years)	83.5 ± 6.1	83.7 ± 5.4	87.2 ± 6.2	86.1 ± 6.4	< 0.001	—
BMI (kg/m^2^)	25.1 ± 4.9	26.3 ± 5.3	26.1 ± 4.1	23.5 ± 4.3	0.001	0.001
S‐Albumin (g/L)	34.1 ± 4.9	35.2 ± 4.8	32.6 ± 4.2	33.2 ± 4.5	0.044	0.047
Barthel Index	62.8 ± 35.7	47.1 ± 27.3	32.6 ± 26.0	39.2 ± 32.0	< 0.001	< 0.001
ADL	5.0 ± 1.7	4.3 ± 1.9	3.0 ± 2.3	3.8 ± 2.1	< 0.001	< 0.001
IADL	5.3 ± 2.7	4.1 ± 2.6	2.0 ± 2.6	3.1 ± 2.6	< 0.001	< 0.001
MNA	24.9 ± 6.0	21.7 ± 7.0	19.6 ± 6.2	21.3 ± 6.6	< 0.001	< 0.001
Charlson Comorbidity Index	6.5 ± 2.5	6.7 ± 2.8	8.0 ± 2.7	7.6 ± 2.6	< 0.001	0.002
Rockwood Clinical Frailty Scale	3.6 ± 1.5	5.2 ± 1.2	5.5 ± 1.2	5.3 ± 1.4	< 0.001	< 0.001

Abbreviations: ADL, Activities of Daily Living Scale; BMI, body mass index; IADL, Instrumental Activities of Daily Living Scale; MNA, Mini Nutritional Assessment; SD, standard deviation; S‐Albumin, serum Albumin; SMI, Skeletal Muscle Index.

**FIGURE 2:**
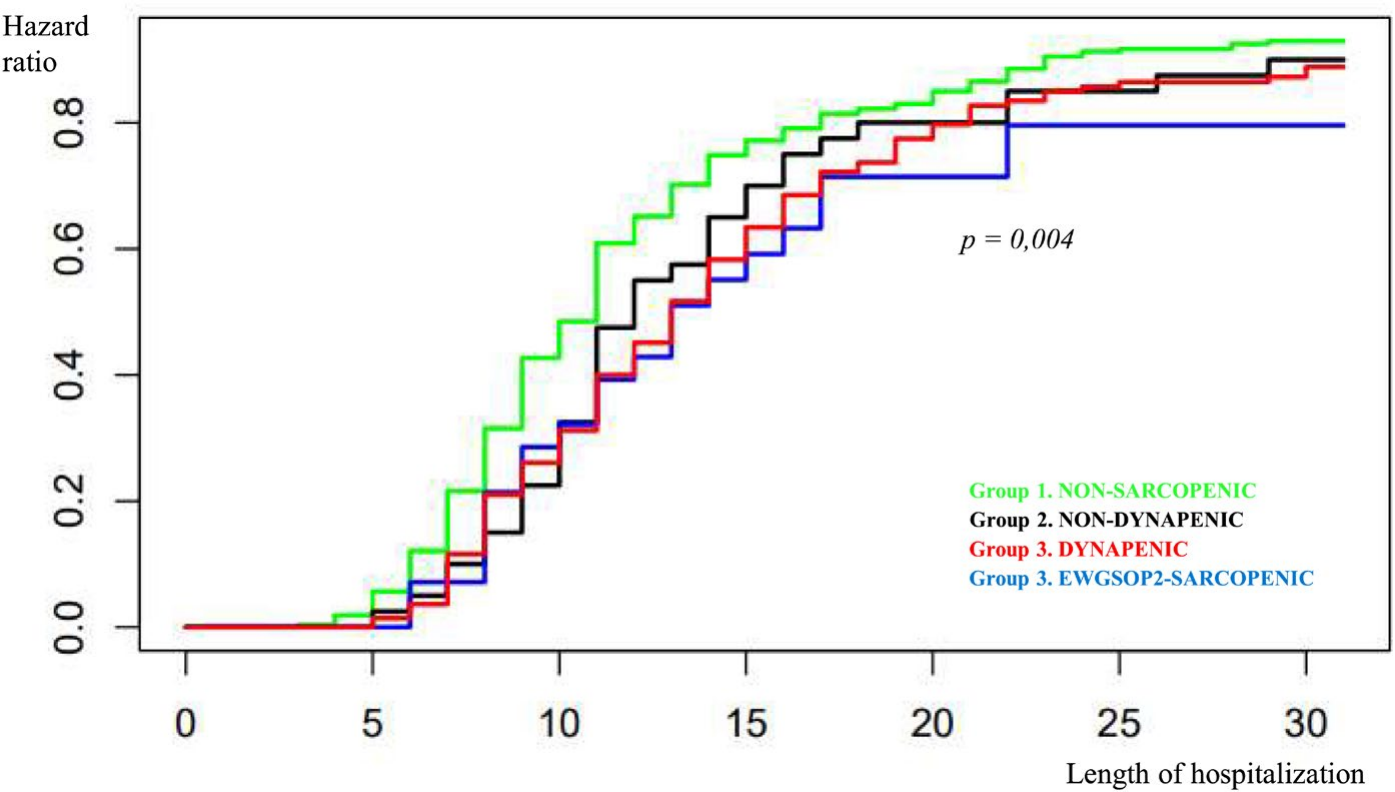
Length of hospitalization in relation to the different groups of old patients stratified according to the EWGSOP2 algorithm for Sarcopenia screening and diagnosis. Group 1 (green). NON‐SARCOPENIC: Negative Screening Test with SARC‐F; Group 2 (black). NON‐DYNAPENIC: Positive Screening Test with SARC‐F, normal handgrip; Group 3 (red). DYNAPENIC: Positive Screening Test with SARC‐F, Dynapenia, normal Skeletal muscle index (SMI); Group 3 (blue). EWGSOP2‐SARCOPENIC: Confirmed Sarcopenia with Positive Screening Test with SARC‐F, Dynapenia and low SMI.

**FIGURE 3:**
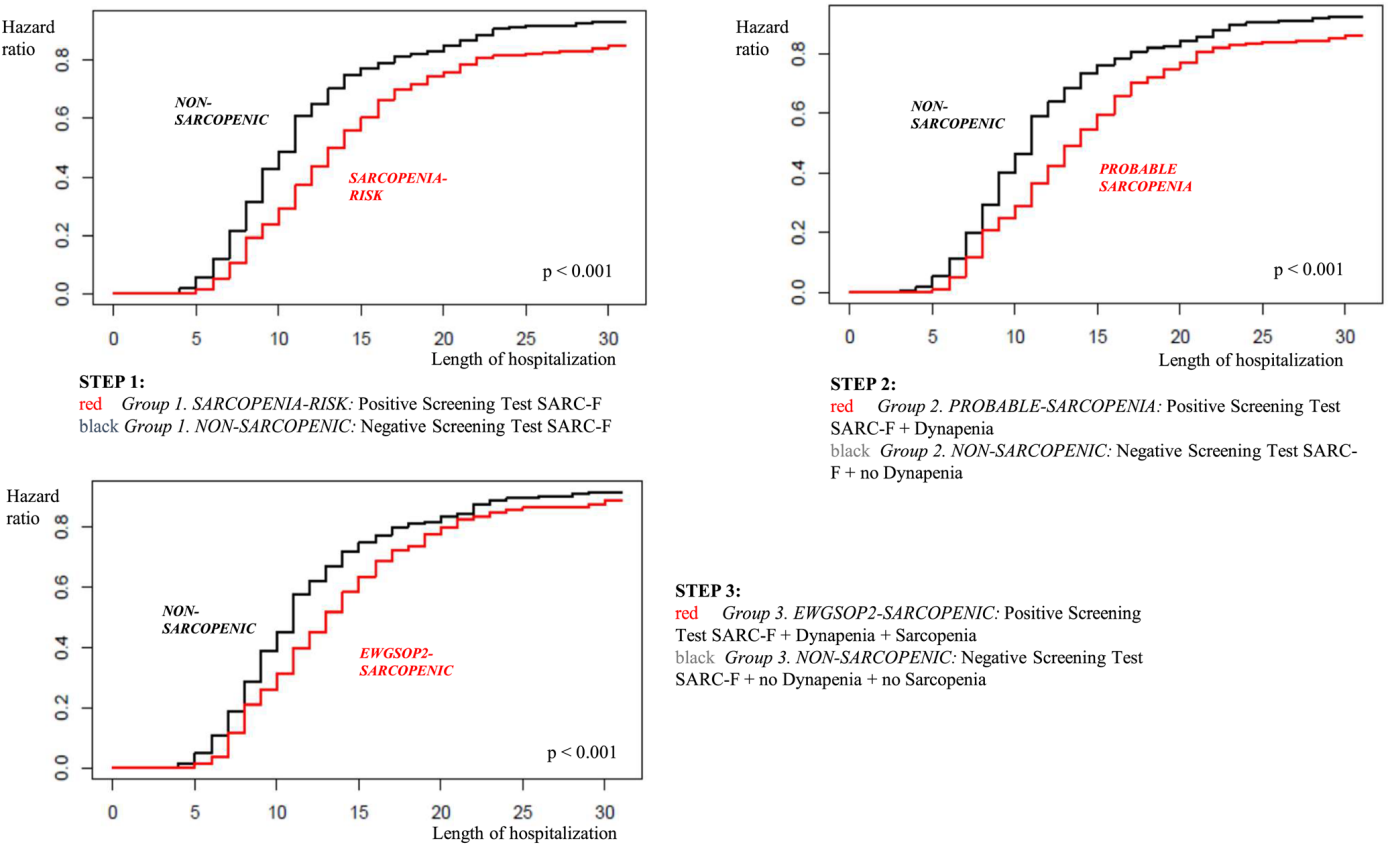
Length of hospitalization in different groups of older patients stratified according to the step‐by‐step application of the EWGSOP2 algorithm for Sarcopenia screening and diagnosis.

We analyzed the main outcomes of the study, that is, the length of hospitalization, the risk of falls and the perceived quality of life, in these different groups of patients stratified accordingly to the EWGSOP2 algorithm (Figures 2 and 3 and Table 2).

**TABLE 2:** Analysis of the main outcomes of the study (risk of falls and quality of life) in relation to the different groups of older hospitalized patients, stratified according to the step‐by‐step application of the EWGSOP2 algorithm for Sarcopenia screening and diagnosis (*n* = 604).

	Group 1. NON‐SARCOPENIC Negative Screening (*n* = 266)	Group 2. NON‐DYNAPENIC Positive Screening, normal handgrip (*n* = 40)	Group 3. DYNAPENIC Dynapenia, normal SMI (*n* = 28)	Group 3. EWGSOP2‐SARCOPENIC Sarcopenia (*n* = 138)	Unadjusted	After age adjustment	After age, MNA, Charlson Index adjustment
	Mean ± SD	Mean ± SD	Mean ± SD	Mean ± SD			
Hendricks Fall Risk Score	3.4 ± 2.7	6.3 ± 3.3	6.3 ± 2.3	5.3 ± 2.8	< 0.001	< 0.001	< 0.001
QoL‐VAS (%)	74.2 ± 19.5	70.3 ± 23.7	62.3 ± 29.8	64.3 ± 24.1	0.001	0.006	0.065

Abbreviations: MNA, Mini Nutritional Assessment; QoL‐VAS, visual analogic scale of quality of life; SD, standard deviation; SMI, Skeletal Muscle Index.

The average length of hospital stay was significantly different (log‐rank test: 13.4; *p* = 0.004) among patients with a negative screening (13.7 days), subjects with a positive screening but no Dynapenia (16.1 days), old patients with Dynapenia (20.4 days) and with confirmed Sarcopenia (17.0) (Figure 2). In particular, the shortest average length of hospitalization was observed in *Group 1. NON‐SARC*, with a median of 11 days; *Group 2. NON*‐DYNAP has a median of 12 days, while *Group 3. DYNAP* and *Group 3. EWGSOP2‐SARC* with confirmed Sarcopenia have a median length of hospital stay of 13 days (Figure 2).

These findings were also confirmed when the length of hospitalization was compared between groups after adjustment for age and for several confounders considered together, such as nutritional status (MNA) and comorbidity (CCI): the difference among the groups remained statistically significant (likelihood ratio test: 10.1; *p* = 0.018) **(data not shown in table)**.

The risk of falls was significantly lower in the group of subjects with a negative screening compared to all the other groups, also after multiple adjustments (Table 2). Finally, the perceived quality of life was significantly higher in the first group of patients with negative SARC‐F values and decreased progressively considering the other groups, especially in patients with confirmed Sarcopenia. However, after multiple adjustments, the difference in quality of life between groups became marginally significant (*p* = 0.065; Table 2).

To further answer the question whether it could be informative to use each single step of the EWGSOP2 algorithm, we divided the patients into negative or positive for each diagnostic step of the EWGSOP2 flowchart (Figure 1), and employing the Cox model, we estimated the corresponding hazard ratio of hospital discharge (negative vs. positive for each assessment) (Figure 3). The average length of hospitalization was 13.7 and 18.8 days in patients with respectively negative and positive SARC‐F values (medians: 11 and 14 days respectively), while the hazard of a faster discharge for negative SARC‐F patients was 1.56 times higher than that of subjects with positive SARC‐F (*p* < 0.001) (Figure 3). When considering subjects with both negative SARC‐F values and normal handgrip strength test compared with those with probable Sarcopenia the average length of hospitalization was, respectively, 14.1 and 18.4 days (medians: 11 and 14 days), while the hazard of a faster discharge for non‐sarcopenic patients was 1.47 times higher than that of probable sarcopenic subjects (*p* < 0.001) (Figure 3). Finally, patients negative to SARC‐F, non‐Dynapenic and non‐Sarcopenic showed an average length of hospitalization of 14.5 days compared to 17.1 days of confirmed Sarcopenic subjects (medians: 11 and 13 days respectively), while the hazard of a faster discharge for non‐sarcopenic patients was 1.3 times higher than that of confirmed sarcopenic subjects (*p* < 0.001) (Figure 3).

Similarly, a significant difference was found for the average risk of fall between patients with positive screening at SARC‐F (6.2 ± 2.8) compared to negative ones (3.4 ± 2.7) (*p* < 0.001) **(data not shown in table)**. A similar result was found when subjects with probable Sarcopenia were compared to negative SARC‐F and non Dynapenic ones (5.9 ± 2.8 vs 3.8 ± 3.0; *p* < 0.001) **(data not shown in table)**. The difference for the average risk of fall between old adults with confirmed Sarcopenia and negative SARC‐F, non‐Dynapenic and non‐sarcopenic ones was also significant (5.3 ± 2.8 vs 4.0 ± 3.0; *p* < 0.001) **(data not shown in table)**.

Finally, a significant difference (*p* < 0.001) was found also for the average perceived quality of life between patients with positive screening at SARC‐F (63.2 ± 24.2) compared to negative ones (74.2 ± 19.5) **(data not shown in table)**. A similar result was found when subjects with probable Sarcopenia were compared to negative SARC‐F and non Dynapenic ones (64.4 ± 23.1 vs 73.8 ± 20.0; *p* < 0.001) **(data not shown in table)**. Similarly, the difference for the average quality of life between old subjects with confirmed Sarcopenia compared to negative SARC‐F, non‐Dynapenic and non‐sarcopenic ones was also significant (64.3 ± 24.1 vs 72.8 ± 21.2; *p* = 0.002) **(data not shown in table)**.

## 4 Discussion

In this study, conducted in a population of old subjects, hospitalized in a Geriatric Acute Care Department, patients with a positive SARC‐F screening questionnaire, in the 85.5% of cases presented also probable Sarcopenia. Among patients with probable Sarcopenia, and with available body composition data, 83.1% were identified as having confirmed Sarcopenia. Length of hospitalization, risk of falls, and quality of life were significantly worse in groups of patients stratified according to the EWGSOP2 algorithm, even after multiple adjustments. Our step‐by‐step analyses also show that each step of the algorithm adds predictivity in sarcopenia‐related clinical adverse outcomes in terms of length of hospitalization, risk of fall, and perceived quality of life.

To date, no specific criteria for diagnosis of Sarcopenia in hospitalized older patients have been proposed and definitions used for community‐dwelling older adults are often applied also in hospitalized patients. From a pathophysiological point of view, it is well accepted that hospitalized patients have a higher risk of Sarcopenia than community‐dwelling subjects, resulting from increased degree of inflammation, loss of mobility and muscle deconditioning, malnutrition and inflammation, all of which are mechanisms that can cause or precipitate age‐related loss of muscle mass and function [2]. Moreover, Sarcopenia is also a risk factor per se of hospitalization [10]. Hospitalization, especially in old patients, can also impact other body composition compartments as total body fat, visceral and subcutaneous fat as well as body water [31, 32].

Our study confirms the EWGSOP2 algorithm as a valid tool for Sarcopenia diagnosis in hospitalized old patients. Our analyses clearly show, for each step of the algorithm, a progressively greater association with adverse outcomes (length of in‐hospital stay, risk of falls, quality of life); all these outcomes were worse in patients with confirmed Sarcopenia, compared to those with probable Sarcopenia, or at risk of Sarcopenia and with normal subjects, independently from several confounding factors.

Our study shows that the EWGSOP2 algorithm may not be feasibly conducted in hospitalized subjects, as there is a number of missing data for each diagnostic step. Measuring muscle mass in hospitalized patients may be challenging: DXA is not always available in all hospitals and often hospitalized patients, with acute illness, cannot be easily moved to obtain a DXA scan [32, 33]. BIA may present several pitfalls in hospitalized subjects as well [33, 34, 35]. Body composition data by BIA measurements can be difficult to interpret due to hydration state, fluid shift toward the extracellular space, presence of inflammation or edema; also, the formula used to infer muscle mass can be different in relation to the characteristics of the population examined [33, 34, 35].

In the recent Delphi consensus of the GLIS, there was a high agreement about the fact that the key aspects of Sarcopenia definition should not vary in relation to the clinical setting [9]. However, the methods to assess these key aspects should change in relation to the characteristics of the patients as well as to the clinical setting considered.

From this point of view, SARC‐F and handgrip strength test can be considered as feasible measures for Sarcopenia in older hospitalized patients, as also confirmed by our data. Moreover, they are generally easily available, low‐cost and low‐time consuming measures.

In particular, the SARC‐F questionnaire has been extensively validated and diffusely adopted as a screening tool for Sarcopenia [36]. SARC‐F has been validated in different clinical settings, in patients with different diseases, and used as a screening tool for Sarcopenia in more than 700 citations [36]. Moreover, SARC‐F is recognized as a useful prognostic indicator not only in adults subjects [37] but also in older hospitalized patients [38], and is associated with an increased risk of in‐hospital mortality [37, 38]. However, this questionnaire presents high specificity but low sensitivity and can identify better severe compared to initial cases of Sarcopenia [39, 40]. From this point of view, in our paper, the presence of a high SARC‐F score and pathological handgrip strength test values seem to increase the specificity of the SARC‐F questionnaire alone, leading to the identification of the patients with confirmed Sarcopenia in 83% of cases.

Similarly, the use of handgrip was widely validated in the diagnosis of Sarcopenia both in hospital and in community dwelling populations and, within the tests assessing physical ability, is one of the most influential tools [36].

Using the step‐by‐step diagnostic algorithm of the EWGSOP2, in our population, we found a prevalence of 22% of subjects with confirmed Sarcopenia, in line with the data of previous studies conducted in hospitalized old subjects [10, 11], showing a prevalence of Sarcopenia ranging from 10% to 40% across studies [10, 11, 41].

In this population study, the application of the step‐by‐step algorithm of the EWGSOP2 may has led to an underestimation of patients with severe Sarcopenia who were probably excluded from the analysis. In fact, in our study, the patients without available dynamometry and body composition data were the most disabled and frail; these patients were older, had lower BMI, MNA scores and albumin values, presented more disability in ADL and IADL, higher scores at the Rockwood Clinical Frailty Scale, and longer hospitalization compared to the other groups. It should be considered that in hospitalized geriatric patients there is also a high prevalence of malnutrition and frailty [42] and we cannot exclude an overlap between Sarcopenia and malnutrition itself [42].

Some limitations of the present study should be acknowledged. First, the results of this study are not generalizable. Other future studies should address this topic in different settings (as rehabilitation or community) or in patients with different clinical characteristics, (i.e., less frail, with lower disability) who might not exhibit the same sarcopenia prevalence or outcomes.

Second, the lack of body composition data and of strength tests in patients with negative screening at the SARC‐F questionnaire could limit the reliability of the analysis, even though it is well‐known that this questionnaire has a high specificity. However, because of its low sensitivity, we could have lost subjects with early sarcopenia, by using the recommended cut‐off of 4 [24]. Future ad hoc studies using lower cut‐off values of SARC‐F, should be performed.

Measurement of fat free mass, not muscle mass, could have limited our findings, even if it must be considered that this limit should be applied to the majority of clinical studies. Further, edema and dehydration, frequently observed in acute ill patients, such as in the present study, are known limiting factors for BIA measurements [35] and further studies by using DXA could be more useful.

## 5 Conclusions

These results show that the EWGSOP2 algorithm is a valid tool even in hospitalized old patients. Our data also show that each step of the algorithm adds predictivity, suggesting that in case of impossibility to perform all evaluations, just SARC‐F plus muscle strength could be useful.

However, more research is needed in finding a wide consensus for a good operational definition of Sarcopenia also in populations of hospitalized frail and disabled old subjects.

## Author Contributions

Conceptualization and design: E.Z., S.U., M.Z. Data acquisition: S.U., A. Giani, A. Gavras. Data analysis: R.M. Data interpretation: E.Z., R.M., M.Z. Drafting manuscript: E.Z., S.U., M.Z. Revising manuscript: F.F., J.A.B. Approving final version of the manuscript: all authors.

## Funding

This work was supported by grants from CORIS (Consorzio per la Ricerca Sanitaria—Regione Veneto).

## Ethics Statement

The study was conducted in accordance with the 1964 Declaration of Helsinki and its later amendments and approved by the Ethics Committee of the University of Verona Hospital (progressive number 1829CESC, approved 28 June 2018, protocol number 46319).

## Conflicts of Interest

The authors declare no conflicts of interest.

## Supporting information


**Supporting Information: Table S1.** General characteristics of the study populations of older patients stratified in different groups according to the step‐by‐step application of the EWGSOP2 algorithm for Sarcopenia screening and diagnosis (*n* = 604).
**Supporting Information: Table S2.** Principal characteristics of the study sample (*n* = 604).
