# Supplementary material for: European Working Group on Sarcopenia in Older People Algorithm: Step‐by‐Step Relation With Length of Hospitalization
Source: J Am Geriatr Soc. 2026 Feb 5;74(3):760–9. doi: 10.1111/jgs.70312 (PMC12968358; doi:10.1111/jgs.70312)
Supplement: Supplementary file 1 — Supporting Information: Table S1. General characteristics of the study populations of older patients stratified in different groups according to the step‐by‐step application of the EWGSOP2 algorithm for Sarcopenia screening and diagnosis (n = 604). Supporting Information: Table S2. Principal characteristics of the study sample (n = 604). [file JGS-74-760-s001.pdf]

Supplementary Table 1. General characteristics of the study populations of older patients stratified in different groups according to the step-by-step application of the EWGSOP2 algorithm for Sarcopenia screening and diagnosis (n=604).

	**Group 1. NON-SARCOPENIC** Negative Screening (n = 266) Mean±S.D.	**Group 2. NON-DYNAPENIC** Positive Screening, normal handgrip (n = 40) Mean±S.D.	**Group 3. DYNAPENIC** Dynapenia, normal SMI (n = 28) Mean±S.D.	**Group 3. EWGSOP2-SARCOPENIC** Sarcopenia (n = 138) Mean±S.D.	**Group 2. HGP-MISSING** Positive Screening, missing handgrip (n = 63) Mean±S.D.	**Group 3. BC-MISSING** Dynapenia, missing body composition (n = 69) Mean±S.D.
Age (years)	83.5±6.1	83.7±5.4	87.2±6.2	86±6.4	87.3±6.0	87.7 ±7.1
BMI (Kg/m^2)	25.1±4.9	25.6±5.3	26±4.1	23.5±4.3	22.8±5.2	23.4±4.8
S-Albumin (g/L)	34.1±4.9	34.3±4.8	35.6±4.2	33.1±4.5	31±5.7	32.8±4.4
Barthel Index	62.8±35.7	47.1±27.3	32.1±26	39.2±32	10.3±15.4	26.2 ±25.1
ADL	4.9±1.7	4.2±1.9	3±2.2	3.8±2.1	1.5±2	2.4±2
IADL	5.3±2.7	4±2.6	2±2.6	3.1±2.6	0.9±2	1.7±2.1
MNA	24.9±6	21.7±7	19.6±6.2	21.3±6.6	16.0±7.1	17.8 ±6.1
Charlson Comorbidity Index	6.5±2.5	6.7±2.8	8±2.7	7.6±2.5	7.4±2.6	7.6 ±2.5
Rockwood Clinical Frailty Scale	3.6±1.4	5±1.2	5.5±1.2	5.3±1.3	6.6±0.8	6.2±0.9

BMI: body mass index; S-Albumin: serum Albumin; ADL: Activities of Daily Living Scale; IADL: Instrumental Activities of Daily Living Scale; MNA: Mini Nutritional Assessment; S.D.: standard deviation; SMI: Skeletal Muscle Index.

Supplementary Table 2. Principal characteristics of the study sample (n=604).

	**Male old patients** (n = 317) Mean±S.D.	**Female old patients** (n = 287) Mean±S.D.	**p value**
Age (years)	84±6.1	86.4±6.6	<0.001
BMI (Kg/m^2)	24.7±4.6	24.1±5.2	0.155
S-Albumin (g/L)	32.9±5.1	34.0±4.7	0.004
Barthel Index score	49.1±37.3	43.0±34.4	0.075
ADL	4.1±2.2	3.6±2.3	0.005
IADL	3.9±2.9	3.4±3.1	0.057
MNA	22.3±6.6	21.5±7.4	0.169
Charlson Comorbidity Index	7.2±2.8	6.9±2.4	0.266
Rockwood Clinical Frailty Scale	4.7±1.7	5.0±1.7	0.033
Lenght of hospitalization (days)	13.1±9.4	13±8.9	0.977
Hendricks Fall Risk Score	5.0±3.2	4.9±3.0	0.774
QoL-VAS (%)	69.7±20.9	66.6±24.7	0.143

BMI: Body mass index; S-Albumin: serum Albumin; ADL: Activities of Daily Living Scale; IADL: Instrumental Activities of Daily Living Scale; MNA: Mini Nutritional Assessment; QoL-VAS: Visual Analogic Scale of Quality of Life; S.D.: standard deviation.
